# Communities’ views, attitudes and recommendations on community-based education of undergraduate Health Sciences students in South Africa: A qualitative study

**DOI:** 10.4102/phcfm.v5i1.456

**Published:** 2013-06-11

**Authors:** Langalibalele H. Mabuza, Paula Diab, Stephen J. Reid, Busisiwe E. Ntuli, Penelope S. Flack, Ratie Mpofu, Priscilla S. Daniels, Tracy-Ann Adonis, Mandisa Cakwe, Mugambi W. Karuguti, Ngkatiseng Molefe

**Affiliations:** 1 Department of Family Medicine and Primary Health Care, University of Limpopo, South Africa; 2 Department of Rural Health, University of KwaZulu-Natal, South Africa; 3 Primary Health Care, University of Cape Town, South Africa; 4 Department of Public Health, University of Limpopo, South Africa; 5 School of Health Sciences, University of KwaZulu-Natal, South Africa; 6 Faculty of Community and Health Sciences, University of the Western Cape, South Africa; 7 Community Engagement Unit, University of the Western Cape, South Africa; 8 Centre for Rural Health, University of KwaZulu-Natal, South Africa

## Abstract

**Background:**

Medical and Health Sciences students in South Africa undertake community-based education (CBE). Health professionals based at host sites are jointly responsible for training of these students in conjunction with university staff. This study explored the communities’ views, attitudes and recommendations regarding CBE undertaken by these students, in order to improve the quality of community support for these programmes.

**Method:**

A qualitative descriptive study was conducted at CBE placement sites of students from the Faculties of Health Sciences of the University of Limpopo (UL), University of KwaZulu-Natal (UKZN) and University of the Western Cape (UWC) during 2010 and 2011. Focus group discussions were held with site facilitators, community leaders and patients, and interviews were audio recorded, transcribed and translated into English where necessary. Data were analysed using NVivo (version 9).

**Findings:**

CBE was seen to benefit communities, students and host institutions as there was perceived improvement of service delivery, better referral to hospitals and reduction of workloads on site staff. CBE was also seen as having potential for recruiting professionals who have better orientation to the area, and for motivating school pupils for a career in health sciences. Students acquired practical skills and gained confidence and experience. Challenges included poor communication between universities and host sites, burden of student teaching on site facilitators, cultural and religious sensitivity of students and language barriers.

**Conclusion:**

The study revealed that communities have an important role to play in the CBE of future health care professionals. CBE activities could be better organised and managed through formalised partnerships.

## Abstrait

**Les points de vue, les attitudes et les recommandations des communautés sur l'enseignement communautaire suivi par des étudiants de premier cycle en Sciences de la santé en Afrique du Sud : Une étude qualitative**

**Contexte:**

En Afrique du Sud, des étudiants en médecine et en sciences de la santé suivent un enseignement communautaire. Des professionnels de la santé basés sur des sites d'accueil partagent la responsabilité de former ces étudiants conjointement avec un personnel universitaire. Cette étude a analysé les points de vue, les attitudes et les recommandations des communautés sur l'enseignement communautaire suivi par ces étudiants, en vue d'améliorer la qualité du soutien communautaire en faveur de ces programmes.

**Méthode:**

Une étude qualitative descriptive a été réalisée en 2010 et 2011 sur les sites d'enseignement communautaire auprès d’étudiants venant des facultés des sciences de la santé de l'université du Limpopo, de l'université du KwaZulu-Natal et de l'université du Cap occidental. Les discussions des groupes de discussion ont été organisées avec des facilitateurs sur le site, des responsables communautaires et des patients, et les entretiens ont été enregistrés, retranscrits et traduits en anglais si nécessaire. Les données ont été analysées en utilisant NVivo (version 9).

**Résultats:**

L'enseignement communautaire est considéré comme bénéficiant aux communautés, aux étudiants et aux institutions d'accueil en raison d'une amélioration perçue des prestations de service, d'une meilleure orientation vers les hôpitaux et d'une réduction de la charge de travail du personnel sur place. On considère également que l'enseignement communautaire a des effets positifs sur le recrutement des professionnels qui seront mieux orientés vers la région, et sur la motivation des élèves à s'engager vers une carrière dans les sciences de la santé. Les étudiants ont acquis des compétences pratiques, une confiance en eux et de l'expérience. Les défis rencontrés sont une faible communication entre les universités et les sites d'accueil, le fardeau de l'enseignement pour les facilitateurs sur place, les sensibilités culturelles et religieuses des étudiants et les barrières de la langue.

**Conclusion:**

L’étude a montré que les communautés ont un rôle important à jouer dans l'enseignement communautaire des futurs professionnels de la santé. Les activités de l'enseignement communautaire pourraient être mieux organisées et gérées par le biais de partenariats officiels.

## Introduction

### Background

Community-based education (CBE) has been defined as ‘a form of instruction where trainees learn professional competencies in a community setting focusing on population groups and also individuals and their everyday problems.’^1^During their training in the community students learn about social and economic aspects of illness; health services in the community and methods of health promotion; working in teams; and frequency and types of problems encountered outside a hospital setting.^1^

A community can be defined geographically or as a social or political construct that can be influenced by its members.^2^ For the purposes of our study the communities were the placement sites where Health Sciences students undertook learning outside the university classroom. Sites included district hospitals, primary health care clinics, non-governmental or non-profit organisations and home-based care. Literature reveals the benefits of this experiential learning to students.^3^ However, some studies have shown that the relationship between communities and centres of higher learning is commonly one-sided, in that communities tend to be passive recipients of services provided by the students.^4, 5^In this study the authors intended to create awareness of what is happening in CBE by exploring views and opinions of both recipients and providers of the service.

CBE has been recognised as potentially influencing students’ career choices as well as addressing community health needs: ‘community based training was identified as the main factor shaping values and attitudes of those who were in favour of rural practice, and were confident and willing to work in rural areas.’^3^ In sub-Saharan Africa there is a human resources crisis of skilled health professionals, with poor retention of new graduates in the health professions, particularly in rural areas.^6, 7^ Hence the imperative to better understand CBE, with a view to improving existing programmes and developing new ones.

This is being addressed by researchers globally and in Africa,^6, 7, 8, 9^ in particular by the likes of Kaye and colleagues^7^ in Uganda, who have looked at both community perspectives and student learning. However, much of the research is discipline-specific and typically refers to CBE in medical or dental education, or focuses on a particular project or context. To our knowledge there has there been no study conducted in South Africa to explore CBE from the community perspective. Studies conducted on CBE focused mainly on services delivery by personnel from institutions of higher learning and student teaching and learning.^8, 9^ The voice of the community where CBE takes place is yet to be heard.

### Aim of the study

This research sought to gain a broader understanding across a range of CBE programmes from multiple Health Sciences disciplines in three universities and different communities. The aim of this study was to explore communities’ views, attitudes and recommendations regarding CBE undertaken by undergraduate Health Sciences students at three South African universities.

### Significance of the study

A number of studies conducted on CBE reported on the benefits which institutions of higher education and training derived from the programme.^5, 6, 7^ There is a paucity of information on the views of communities (including community leaders, site facilitators and patients) where CBE has been conducted. This paper sought to bridge that gap.

## Research method and design

### Design sampling

An exploratory qualitative study was conducted at three South African universities, the University of Limpopo (UL) (Medunsa Campus), University of KwaZulu-Natal (UKZN) and University of the Western Cape (UWC).These three universities are members of Collaboration for Health Equity through Education and Research (CHEER), and showed interest in exploring this topic. CHEER is a collaboration of academics involved in CBE which has been in existence since 2003, and includes members from nine Health Sciences faculties in South Africa: UKZN, University of the Free State, Walter Sisulu University, Stellenbosch University, University of the Witwatersrand, University of Cape Town, UL, UWC and University of Pretoria.

Facilities were selected as research sites based on the CBE programmes in the Health Sciences faculties/schools at the respective universities; sites were selected through purposive sampling to include both urban and rural sites (UWC's were mainly rural), as seen in Table 1.

**TABLE 1:** Placement sites for each of the three participating universities.

University	Placement site
University of Limpopo	Phedisong 4 Clinic (Gauteng Province) (urban)
	Rustenburg Clinic (North-West Province) (urban)
	KT Motubatsi Clinic (Gauteng Province) (urban)
	KwaMhlanga Clinic (Mpumalanga Province) (rural)
University of KwaZulu-Natal	Beatrice Clinic (urban)
	R.K. Khan Hospital (urban)
	Murchison Hospital (rural)
	Eshowe Hospital (rural)
University of the Western Cape	Grabouw Clinic (rural)
	Genadendal 1 Clinic (rural)
	Genadendal 2 Clinic (rural)

The sites selected across the six provinces were those where CBE programmes for nursing, occupational therapy, speech and language therapy, audiology, pharmacy and physiotherapy as well as for medical students took place for the three universities. Sites not involved in CBE and those where permission to conduct the study was not obtained were excluded.

### Procedures

Community views, attitudes and recommendations were explored through three groups of participants at each site. The first group was made up of community leaders in charge of the training facilities where students were posted; they were the recognised leadership in the respective communities, such as ward councillors, church leaders, and hospital board members. The community leaders were not direct recipients of the services in the way that patients were, but typically were gatekeepers for community access. The second group comprised community members involved in CBE facilitation, referred to here as site facilitators, who were supervisors of student training at the host institution. The third group comprised community members who were patients or clients interacting with students, in other words recipients of the service provided by the students.

### Materials

Focus group interviews were conducted at each of the selected sites, comprising an average of five participants each, with the three participant groups interviewed separately by the CHEER members of each university. Purposive sampling of participants was used to ensure richness of the required information, as recommended by Maree.^10^ Discussions were conducted within the health care facilities which were the student training sites. An agreed interview schedule was used as a guide for the focus group interviews. All three groups per site visited were asked about their views on the benefits of the programme, on the health care students provided and recommendations for future programmes. Specific questions to site facilitators focused on the university brief on programme facilitation; specific questions to the community leaders were about their awareness of and role in the programme. Specific questions to patients explored their awareness of the fact that they were being attended to by health care students and their perceptions of that interaction.

### Data analysis

CHEER members from each of the three participating universities were involved in coding of the transcripts. All interviews were audio recorded, and supplemented by field notes which were used during transcription when audibility was compromised. Recorded interviews were transcribed verbatim, and when the language of the interview was the local community language such as Setswana, Sepedi, IsiZulu, IsiXhosa or Afrikaans, transcripts were translated into English. Accuracy of the English translations was verified through cross-reference to the recordings.

Content analysis of the data was conducted. The CHEER representative and research intern from each university (six members) plus the national CHEER coordinator conducted more than one reading of the transcripts to identify themes as per the interview guide. A final round of data analysis took place in a peer-debriefing meeting of all collaborating universities, where consensus was reached on major themes and subthemes. To ensure neutrality one collaborating member who did not take part in coding of transcripts led the peer-debriefing meeting. Data analysis was aided by use of NVivo (version 9).

Various strategies were employed to ensure trustworthiness: the researchers interviewed participants in their local languages, audio recorded the interviews and made verbatim transcriptions. Use of data from the transcriptions and field notes ensured triangulation.^11, 12^

## Ethical considerations

Ethical approval was obtained from the Medunsa Research and Ethics Committee of the UL (MREC/M/20/2010:IR), Humanities and Social Sciences Research Ethics Committee of UKZN(HSS/0210/010) and the Research Ethics Committee of UWC (registration number 10/7/15). Permission to conduct the study was obtained from senior management at the various study sites in each province as well as from the relevant authorities within Provincial Department of Health boards. All participants signed a consent form and were informed about their participation and that they could withdraw at any point in the process.

## Results

The findings reported are substantiated by adding quotes from the three participant groups. Responses from all three groups fall into two broad categories: the benefits of CBE and the challenges which potentially pose a threat to the respective programmes, with recommendations provided by participants.

### Benefits of CBE

The benefits of CBE were reported for the community at large, the site facilitators and the students. Although the students were not included as participants, benefits to the students were reported by all three participant groups, the primary benefit for all being improved service delivery. Students were also seen to be able to assist with referrals to hospital, diminishing the need for patient referrals from the clinics and thus alleviating congestion.

#### Site facilitators

This site facilitator (SF) confirmed the benefit of **s**tudents being able to make referrals:

‘Now patients who are seen by the [*student*] doctors here do not [*need*] to go to the hospital … we are happy that our pensioners … will not be going to the hospital but will be seen here’ (SF, UL)

Another perhaps indirect benefit to communities is the potential for recruiting students who are already familiar with the context once they are qualified:

‘I think by exposing them to a rural area they get interested in that field of work and as they have been here it might help us get the very same students to come here’ (SF, UKZN)

Through the exposure students were perceived to acquire clinical skills and gain confidence, making them better prepared for future clinical training and practice:

‘… and you could see the difference when she first came in and when she left, she was much [*more*] confident and improved clinically as well …’ (SF, UKZN)

Exposing medical students to rural contexts was seen to benefit them by providing an alternative view for future career pathing, in that in such settings competency in general medicine was viewed as important, as opposed to specialisation which was often emphasised in tertiary training centres:

‘I think one of the things [*that*] are important is that we are also building our doctors for the future, we want to show people that there is another career alternative except for specialising because at medical school they get taught basically idealism: you have to be a gynaecologist or surgeon, that is the main thing they see the whole time. Only when you come here you cannot say this is not my problem, because everything is your problem’ (SF, UKZN)

The site facilitators felt that the programme offered the students good practical experience. There was reciprocity of learning and exchange of knowledge between students and themselves:

‘What they really did there inspired me as well, I also learnt a bit’ (SF, UWC)

‘I think their coming here is very important because whatever there is that is difficult for the [*nursing*] sisters gets referred to them’ (SF, UL)

‘…on the other hand, they teach us something.’ (SF, UL)

In addition to the students’ own experiences, site facilitators enlisted the students’ services to motivate school pupils for a career in the health care sector:

‘…because like the group that worked with [*me*], we had to go out to school and talk to children about it. I think it should have a positive impact on the society.’ (SF, UKZN)

#### Community leaders

The community leaders (CL) viewed the programme as beneficial to the community as well as to the students. Compared to qualified professionals, medical students were perceived to be more dedicated in their interaction with patients, and viewed as helping to ease the workload:

‘These [*student*] doctors are so helpful because there is a shortage of doctors’ (CL, UKZN site)

Community leaders perceived the students as playing a role above that of nursing sisters, and rendering services that could be equated to those of qualified doctors:

‘… people need a doctor urgently because the doctor and sister do different things. That is the importance of doctors, and that is why they need to be in the clinic.’ (CL, UL site)

‘… in my view [*student*] doctors have more knowledge than what we have in the clinic. There are some problems that the sisters cannot solve’ (CL, UL site)

The community leaders perceived the students as adding service value:

‘So, I think the presence of the students in the community gives more value to the community project [*in Genadendal*]’ (CL, UWC site)

‘I know they [students] are very involved with the nurses, and the nurses see the benefit of interacting with them in the clinic’ (CL, UWC site)

‘The students have taught the community to do sewing and knitting. Furthermore, they have taught the mothers to generate own income through gardening in their own backyard’ (CL, UWC site)

#### Community members

Patients (community members [CM]) viewed the medical students as more knowledgeable on the medicines they prescribed and more thorough in the consultation than the facility nurses. Patients therefore preferred to be seen by the students:

‘… but with them, they gave me medication and I saw a big difference.’ (CM, UL site)

‘… immediately as you arrive at the OPD, the [*student*] doctors attend to you.’ (CM, UKZN site)

‘… they [*should*] let them come more often like perhaps three times a quarter …, that would be helpful’ (CM, UWC site)

They were also seen as helping to reduce the clinic queues, thereby improving quality of service. Unlike the already qualified professionals, community members viewed the students as being patient with clients:

‘I was satisfied the way they checked me, I was satisfied because they did not rush any one, they took a long time checking me…’ (CM, UL site)

‘The way they check, they check in the mouth, the ears, the eyes, my back I have never met a sister who checked me that way’ (CM, UL site)

### Challenges

Challenges highlighted included poor communication between universities and host sites, and the burden on site facilitators of student teaching over and above service delivery. Other challenges related to the CBE programme structure, cultural and religious sensitivity of the students, language barriers and inconsistencies in the community's attitude towards students.

Communication between the universities and site facilitators as well as between sites and community members appeared to have been a significant challenge.

Some site facilitators reported that universities, for the most part, did not formally inform them of the student programme, and that they were not given guidance as to course structure and content. They were unaware of the duration of stay of each group of students, and were not fully briefed on their responsibility to teach the students:

‘… so I think there should be more information, clearer information because the people may not understand it properly so we [*need*] to share more information with each other [*university and student placement site*] so that everyone is on the same page…’ (SF, UWC)

‘… I want more information from the university, students or someone who must

explain it to me what the project is all about…’ (SF, UWC)

‘… we are not formally told that [*these are*] your students, you are supposed to teach them 1, 2, 3. They [students] just introduce them[*-selves*] to us – that's all’ (SF, UL)

‘I think it [*teaching of students*] happens a bit by default because I have been involved and interested in the long time – I am a family physician and probably by default it has been pushed into me because I like doing it.’ (SF, UKZN)

Communication seemed to follow the tradition that students were sent to placement sites year after year, often as a result of individual relationships:

‘I think it is almost unspoken because it develops over a longtime, I think the students have been coming here as long as the rotations have been going on, probably for more than ten years … so I think [*that*] is what is expected of everybody …’ (SF, UKZN)

Although most community members (leaders and patients) felt the presence of students resulted in much better service delivery, some site facilitators mentioned that students were a burden in the clinics, especially when it was busy and patients needed to be seen fast enough to be able to access transport back home:

‘It is a great learning opportunity for the students but it can be quite a burden on the hosting institution, in a sense that we are busy and our efficiency is impeded by the fact that we have a teaching responsibility as well…’ (SF, UKZN)

Site facilitators also expressed their concerns regarding lack of material and human resources, which affected student training. They indicated that they could not cope with student supervision alone and would have liked to have university lecturers assisting with facilitation:

‘…they should send more facilitators [from the university] or maybe a mentor, they should maybe make more mentors for the student because sometimes it becomes a problem for the sister’ (SF, UL)

‘I see the university as an academic institution with lots of resources … it should not be so difficult for the university to link up with our [*hospital*] internet system …’ (SF, UKZN)

Various community members appreciated the presence and role of the students in the community health care facilities. However, in some contexts the services offered by students provoked an inflow of patients from other communities not usually covered by that clinic, hence paradoxically increasing the workload:

‘… Although their work is good, their coming caused us a problem because the surrounding areas like Hebron and Mabopane now come here …’ (SF, UL)

Whilst CBE activities were highly valued by the communities, there were challenges in the way they were structured. The site facilitators were of the view that student exposure to practical experience started too late into their course:

‘At least we must have those who are in the second year, third year just like that, just to be exposed earlier because they are doing theory most of the time. If they can mix theory with practical at least to get an idea of what is happening, because you find out that they don't even know how to take Hb [*haemoglobin*] or blood glucose and they are doing their sixth year …’ (SF, UL)

Members of communities explained various forms of cultural and religious challenges that they and the students faced in their interaction. Specific cultural practices and religious doctrine may, for example, impact on the manner in which a physical examination of a patient is conducted, or behaviour towards an elderly person:

‘… what he said is right because that is what we deal with Muslim females, they will sit there and when the nurse comes she will say she wants to see a Muslim doctor and they must be aware of that too …’ (SF, UWC)

‘… say like if it is a *gogo* [*granny*] who has a gynaecological problem and is looking at this one is too young, “No sister I want to be seen by you, I'm too old to be seen by a young one”… they take it like cultural things you know, a young one must not see the private parts of an elder …’ (SF, UKZN)

The lack of communication made community leaders feel undermined, as they were not in a position to inform the communities they were representing:

‘Yes, S said it well, but you see if things are happening in the clinic and you are not informed as the link between the community and the clinic, one of the thing is that we feel undermined because we can't even respond to the communities … [*to*] tell them not to be surprised’ (CL, UL site)

‘They [*students*] should be introduced especially in the clinics … I need to be honest that they are not introduced in the communities’ (CL, UKZN site)

‘Yes, in the beginning the students held meetings with me and my colleague, they had beautiful ideas to help the community. But afterwards, they organised meetings alone and that did not seem to work’ (CL, UWC site)

Communication with the community members as patients was only hampered by the language barrier:

‘I was praying to get someone who speaks IsiZulu and was lucky so that we could understand each other’ (CM, UKZN site)

‘The biggest barrier is the language’ (CM, UKZN site)

### Community attitude towards students

The majority of responses towards students’ behaviour were positive. The students were referred to by both patients and site facilitators as being humble, friendly, conscientious and considerate.

Some site facilitators also remarked on the lack of a sense of responsibility amongst some students:

‘… They will just sit and talk, sometimes they just wait for transport laughing and talking at the same time and then they want your signature …’ (SF, UL)

Furthermore, only very few students were deemed exceptional, ‘who will be confident and competent to go ahead and run the clinic by themselves with minimal supervision’ (SF, UKZN).

Community leaders were well disposed towards the students, viewing them as potential substitutes for retiring doctors:

‘The programme should continue because even the qualified ones, the time will come for them to retire so it is good when the youth study’ (CL, UKZN site)

In some instances community members expressed concerns regarding students’ ability to treat patients:

‘What I heard is that some patients were saying they are sending us students who are going to give us wrong treatment …’ (CM, UL site)

### Recommendations

All three groups of respondents made recommendations relating to communication with the university, facilitation resources, programme structure and students’ behaviour.

Most recommendations came from site facilitators. They recommended that the university provide them with a curriculum guide, including what was expected in terms of student facilitation; also that there be joint planning between the community and universities on curriculum development and student placement, perhaps through formalisation of the partnership to enable resource sharing:

‘Like I said, we need to be involved and know about each other. If I knew what they really wanted to do and if they could tell me what they expect of me and we network, I think it would be wonderful’ (SF, UWC)

‘We don't know if what we are doing is good for them’ (SF, UL)

‘I think maybe when the students come here, if they can bring maybe a programme, something in written form to say they are lacking in this section …’ (SF, UL)

There was also a recommendation that students be supported through mentors from universities to capacitate service delivery at the training sites, and for students to work in pairs to support one another:

‘… they should send more facilitators [*from the university*]; they should maybe make more mentors for the student because sometimes it becomes a problem for the sister because immediately the queue is slow then the patients start fighting and it ends up not being nice …’ (SF, UKZN)

‘When they are paired, I should think it is easy for them to ask each other, support system …’ (SF, UL)

Over and above mentorship, the site facilitators recommended support in the form of resources, new guidelines and new thinking. This would be an encouragement for the site facilitators to assist the students:

‘I see the university as an academic institution with lots of resources … it should not be so difficult for the university to link up with our [*hospital*] Internet system …’ (SF, UKZN)

Recommendations about programme structure included that CBE should be introduced early in the students’ training, preferably at first-year level, as this would encourage a linkage of theory and practice. That would also increase the students’ self-confidence and enable them to learn to work independently.

The community also recommended an increase in the duration of students’ stay at placement sites to maximise mutual benefit, and that student learning activities be scheduled during ‘off-peak’ hours of a facility:

‘Maybe if they can lengthen the time and if they can spend a little more time, they will get more out of it … because two weeks is short’ (SF, UKZN)

‘… during the night we've got plenty of time to teach them …’ (SF, UL)

‘I think during the week, the students aren't full but I think on weekends they must be more involved in different activities in the community and not just keep office hours 8 am to 4 pm’ (SF, UWC)

The need for continuity of service rendered by the students was expressed in various ways by site facilitators, community leaders and patients:

‘… for the nursing students they are here for seven weeks but they only have one day out of the week that they are working on the project, so at the end of it you are looking at only seven days on which they have to do a project’ (SF, UWC)

The community remarked that students started projects, for example on quality improvement, but could not stay long enough to complete them:

‘… but after they have gone, it is no longer there … there is nothing happening regarding the improvement they have made.’ (SF, UKZN)

Community leaders remarked on the language barrier between students and patients. They recommended basic knowledge of the local languages, such as IsiZulu, Afrikaans, SePedi, isiXhosa and others by students:

‘They should include IsiZulu during their training because they get paid to work in the communities, I cannot be the interpreter’ (CL, UKZN site)

‘… we need these doctors indeed but they should have basics of IsiZulu, it is hurting to see that a child cannot speak IsiZulu’ (CL, UKZN site)

Community members appreciated the continuity of care by a particular student (‘doctor’), which they hoped for when coming for a consultation:

‘… when you come to hospital you know you will get Dr so-and-so but on arrival that doctor is not there, maybe the doctor had helped [*you*] before, but when the doctor is no longer there you just do not know whom to ask’ (CM, UKZN site)

## Discussion

The study reveals that CBE was seen to benefit the community, students and host institutions, although balancing service delivery with supervision was a challenge. There was poor communication between the universities and respective health care institutions. Students were generally well received by the host institutions, and in most instances were viewed as an asset to the community. Despite the challenges, communities offered valuable comments on how the programme could be improved.

### Benefits of CBE

The primary benefit for all was improved service delivery. For the site facilitators, benefits related to reduced workload; for the patients, to shorter queues and waiting times; and for the students, to the experience they gained. Many of the benefits were interlinked and/or reciprocal; for example, as the students gained experience, they were able to support the staff by sharing the workload, patient care was improved due to extra time that could be spent, and as students applied theory to practice they brought new knowledge to site facilitators. This situation has been described by Bean^13^ as ‘win-win’, as service is provided to the community whilst students get an ‘outstanding educational and professional experience’. The main focus of learning for the students was on learning practical skills, one of the main features of CBE.^14^ In our study the communities were noted to benefit from ‘faster queues’ and alleviation of the ‘shortage of doctors’.

Community leaders felt that teaching professional behaviour to students was one of the most important tasks of student site facilitators. Hood^15^ is of the view that teaching professional behaviour is one of the objectives of CBE. Most responses on students’ behaviour were positive, which enabled patients to feel they could be open and honest and, with few exceptions, patients were satisfied with the services students rendered.

### Challenges

Collaboration between communities and universities has been used to facilitate knowledge sharing in a variety of sectors, including the health sector.^16^ However, in this study communities reported that they were not informed of the student training programme at the training sites. The lack of communication made community leaders feel undermined, as they were not in a position to share information with the communities they represented. The site facilitators also felt that students’ community entry should involve community leadership.

The negative effect of lack of communication with community leaders and therefore with the community at large was the missed opportunity for shared ownership of the programme. Joint ownership with communities has been found to play a significant role in student learning in ‘the community as a classroom’.^17^ In this study the concern of community leaders indicated the need for a dialogic relationship between the two partners.

Site facilitators were not formally informed on the student programme, and were not given guidance as to course content. They indicated that they were not aware of the duration of stay of each group of students coming to their sites, they were also not aware of the academic levels of the students they had to facilitate. Neither were they aware of their responsibility to teach the students. This could negatively affect student learning, since it has been shown that staff enthusiasm for student presence enhances student learning experience.^18^ It has also been shown that site facilitators prefer coordinated communication between the universities and placement sites both prior to and during clinical placement.^19^

Regarding the students’ placement, there was a disproportionate allocation of students to site facilitators, which created a teaching burden. There was also poor communication between the university and placement sites which impacted on proper planning, e.g. provision of teaching space and allocation of manageable student numbers to site facilitators. This was also found by Kristina, Majoor and Van der Vleuten,^20^ who found ‘few medical schools involved the community in the process of programme planning and evaluation’, resulting in imbalance in student allocation to teaching sites.

Communities expressed concern about the lack of resources, indicating that staff members, equipment to suit different disciplines and budgets were insufficient. They also expressed a desire to be equipped with more formal instruction from the university on how to teach and interact with the students.^6, 21^

Whilst this was regarded as a challenge, it was simultaneously providing future health care professionals with experience of the context of service delivery within marginalised rural areas, and cultivating understanding of the barriers to and social determinants of health in the rural context.^22^ Bruning and colleagues^23^ state that in poorly resourced settings students experience ‘real world’ lessons and acquire skills that complement classroom teaching, introduce civic responsibility and provide leadership experiences.

Language and cultural competence were seen as a challenge as they play a vital role in professional interaction between students and patients.^6^ Some students were working in cross-cultural settings where they had difficulties because of their lack of understanding of local languages. Where nurses assisted with interpretation during the consultation, this increased their workload and became potentially counterproductive.

## Limitations of the study

It is acknowledged that the researchers were all healthcare professionals, which may have influenced the subjectivity of the findings. It is also acknowledged that the findings included views and opinions of participants about CBE which are somewhat limited in their generalisability. However, qualitative research designs are often not generalisable although they offer opportunities of ‘transferability where the burden of demonstrating the applicability of one set of findings to another context rest more with the investigator who would make the transfer, than with the original investigator’.^24^ Furthermore, potential participants who could have provided rich information did not consent to participate and were excluded.

## Recommendations

The main recommendations from this study are to do with community involvement in curriculum development and site planning; improved communication; student orientation to communities prior to placement; linking practice and theory from the first year of study; and culture and language.

Improving communication is regarded as the foundation to establishing partnerships. The manner in which this could be addressed includes making students’ curricula available to site facilitators to enable guided student learning, and for the community and academic institution to engage in joint planning in all matters relating to student placement. It is strongly recommended that the partnership be formalised to encourage sharing of resources. The community recognised that their contribution was not merely an input to the project but formed the basis upon which the project would operate. Meyer and colleagues^25^assert that community- academic partnerships in training of medical professionals can induce a paradigm shift such that physicians view the community as a teaching resource and partner rather than a passive recipient of services or just a placement site.

The Health Sciences faculties have to take the recommendations made by the community seriously. The communities felt that CBE activities should be introduced early in the students’ undergraduate training, preferably at first-year level, as this would link theory and practice. The communities were also of the view that early exposure to practice would increase students’ self-confidence and enable them to work independently. Dornan and co-workers^26^ found that broadening student experiences within community settings was more beneficial if introduced earlier, and the importance of practical experience in the community is confirmed by Mudarikwa et al.^22^

Although we did not find religion and culture to be obstacles to students’ community participation, there is a need for students to have the basics of the community languages. Mbalinda et al.^27^advise that language and cultural understanding are crucial in the development and maintenance of strong relationships between students and the communities they are training in.

The study recommendations are based on those made by the community members. By improving communication with the host institutions and involving communities in planning of the CBE curriculum, CBE activities could be better tailored to suit the needs of the host health care institutions and academic training institutions. Introducing CBE early in the curriculum is strongly recommended, with a continued and sustained programme throughout the undergraduate curriculum in order to obtain maximum benefit for students and communities alike.

It is also recommended that an in-depth exploration of each institutional approach and CBE programme offered could highlight and give insight into some similarities and differences of implementation amongst the three institutions.

## Conclusion

The study reveals that communities have an important role to play in educating future health care professionals, and indicate a commitment to facilitating that process. Students are viewed as vital members of the health care team by all members of the community. Site facilitators see their role as mentoring and guiding students at community sites as well as introducing them to the community. Communities themselves see that they have potential to encourage students to return to work within their placement settings, and acknowledge the positive effect this would have in the long term. However, there is a need for more structure in CBE activities through effective communication and formalised partnerships.
